# ‘It's good to have the knowledge and the confidence’: Mental health student nurses' views of a medication workshop

**DOI:** 10.1111/inm.13012

**Published:** 2022-05-13

**Authors:** John Goodwin, Sheila White, Maria O’Malley, Emma Hurley, Aine O’Donovan, Peter Kelly

**Affiliations:** ^1^ School of Nursing and Midwifery University College Cork Cork Ireland; ^2^ Cork/Kerry Mental Health Services Cork Ireland

**Keywords:** education, internship, medication management, mental health, pharmacology, students

## Abstract

The administration of medication in a safe manner is a key nursing role and nursing regulatory bodies mandate that it is part of undergraduate training. However, it has been noted that qualified nursing staff are dissatisfied with the knowledge demonstrated by students. As such, a 2‐day evidence‐based workshop on medication management for final‐year nursing students was facilitated to address knowledge deficits. Content was delivered by academics and practicing nurses. The aims of this study were to evaluate internship mental health student nurses' experiences of a 2‐day medication management workshop. A qualitative descriptive design was adopted. Three focus group interviews were held with student nurses who partook in the workshop. Data were analysed using reflexive thematic analysis. Three themes were identified: Developing Confidence around Medication Management, Reflections on Learning Gained from the Workshop, and Enhancing Awareness and Vigilance of Medication Errors. Overall, participation in the workshop was identified as having a positive impact on students' knowledge, competence, and confidence in relation to becoming a safe practitioner. Students reported that the timing of the workshop – during internship – was appropriate for their knowledge needs. Findings indicate that the right amount of information was delivered by the right people, at the right time, in the right way. This study has highlighted the positive impact of a 2‐day workshop delivered in the final year of mental health student nurse training, on their confidence and competence in the area of medication management. It provides some insight into how a practical collaborative approach to this type of education between academics and clinicians can help students bridge the theory–practice gap.

## 1 INTRODUCTION

The safe administration of medication is a key nursing role. Mental health nurses spend a substantial amount of time on medication management, with medication errors well documented as being common yet avoidable (Donaldson *et al.,* 2017; Morrison et al., 2018). Building on previous research that has explored undergraduate mental health nursing medication education from the staff nurse perspective (Goodwin et al. 2019a), a 2‐day workshop was developed and facilitated with a group of fourth‐year student mental health nurses, midway through their 9‐month internship (final‐year placement). The workshop was subsequently evaluated to elucidate the students' views, and the impact of the workshop on their needs and practice.

Nurses spend a significant, though variable, amount of time administering medication. Studies have uncovered between 20% and 40% of time spent in the preparation or administration of medication (Armitage & Knapman, 2003; Holmqvist *et al.,* 2018). Medication errors are substantial, the majority of which occur at the point of administration, by registered or student nurses (Cottney & Innes, 2015; Dehven *et al.,* 2021). Factors influencing medication errors include lack of training, inadequate drug knowledge and experience, inadequate knowledge of the service user, and the inadequate perception of risk (Goodwin *et al.,* 2019a; Mariani *et al.,* 2017; World Health Organisation, 2016). These factors ultimately impact service user care, resulting in injury and avoidable harm (Leahy, 2017; World Health Organisation, 2016). To enhance medication safety, all nurses need to know how medications affect service user symptoms, possible risks, or adverse reactions (Leahy, 2017). To achieve this, education and training relating to systems and practices are vital throughout the student nurse's journey and as they begin their practice as registered nurses (World Health Organisation, 2017). Professional nurse bodies endorse this, stating that student nurses must receive adequate education, training, experience, support, and supervision in medication safety and administration (National Council of State Boards of Nursing, 2022; Nursing and Midwifery Board of Ireland, 2020).

Developmental models emphasize the need to develop competence through exposure to skills, participation, and identification, to arrive at internalizing and dissemination of skills and knowledge, thus moving from novice to expert (Benner *et al.,* 2010; Steinaker & Bell, 1979). Nursing students must first acquire knowledge of medications and safe preparation and administration, demonstrate the ability to retain this learning, before translating knowledge and skills into clinical practice (Chuang & Tsao, 2013). Traditionally, this process has been achieved by combining theoretical knowledge delivered in academic settings and real‐world learning in the clinical environment (Coyne *et al.,* 2013). Whilst it is accepted that learning though experience is critical (Mollart et al., 2021; Palominos et al., 2019) and as such, clinical placements are essential learning environments to gain knowledge and skills (Betts, 2016), there remains a gap between theory and practice (Goodwin et al., 2019b; Murray et al., 2019) and a recognition that greater collaboration is needed between academic and clinical settings to strengthen students' skills and knowledge as they progress towards registration (Goodwin *et al.,* 2019a; Musafifi & Daniels, 2020).

In a study of registered nurses' perceptions of the delivery of medication education to undergraduate mental health nurses, Goodwin (2019a) found that students had difficulties interpreting medication charts and were unable to provide information about medication to service users. Participants in this study recommended a practical approach to education, such as inviting clinical staff to present current developments in medication management. Findings from this study informed the development of a 2‐day medication management workshop, facilitated with fourth‐year student nurses, in a university setting in the south of Ireland. Previous research on medication workshops indicates positive outcomes for students of medicine and pharmacy (Bridgeman et al., 2018; Katoue & Haqan, 2013); less is known about how this approach to medication education impacts on mental health nursing students. Therefore, the aim of this study was to evaluate mental health student nurses' views of a medication workshop, the impact of the workshop on their needs and practice, and to uncover any potential changes or improvements to the workshop.

## 2 METHODS

### 2.1 Design

This study adopted a qualitative descriptive approach. ‘Qualitative descriptive studies offer a comprehensive summary of an event in the everyday terms of those events' (Sandelowski 2000, p. 336). Data (transcripts from individual or group interviews) focus on the where, what, why, and when of phenomena. The resultant descriptive narrative offers a low‐inference interpretation of data that is close to the accounts proffered by participants (Sandelowski 2000).

### 2.2 Intervention description

The interactive workshop was facilitated by academic and clinical staff with backgrounds in nursing and pharmacy, with a focus on the various forms of medications, their purpose, and side effects. A full account of the workshop content is outlined in Table 1.

**TABLE 1:** Workshop content

Day 1	Day 2
**9.30–10.30** **Medication Image Identification:** Students were presented with a PowerPoint presentation featuring images of medications. Students were instructed to identify the medications' generic and trade names and doses. At various intervals, students were asked questions about the medication they had identified	**9.30–10.30** **‘Spot the Error’ (Case Study):** A case study was presented to students, focusing on breakfast, lunch, and dinner medication rounds, with errors deliberately inserted. Students were instructed to identify these errors
**10.30–11.00** Morning break	**10.30–11.00** Morning break
**11.00–12.00** **Intramuscular (IM) Injection Technique:** An overview of IM injections and guidance on current best practice guidelines on administration was given using PowerPoint. There was then a demonstration of IM technique with a question‐and‐answer section afterwards	**11.00–12.00** **‘Spot the Error’ (Medication chart):** Medication charts with deliberate errors were presented to students. Students were instructed to identify these errors. Time was allowed at the end of this session for students to ask questions about interpretation of the medication chart
**12.00–13.00** **Overview of Medication Side effects:** A PowerPoint presentation on common side effects of medication and ways to alleviate these was given to students. Extrapyramidal side effects of antipsychotics and neuroleptic malignant syndrome were then discussed. Time was allocated for questions following the presentation	**12.00–13.00** **Student Presentation 1:** Each student presented for 3–4 min on a medication they had been assigned in advance of the workshop. Time was allowed at the end of each presentation for questions
**13.00–14.00** Lunch	**13.00–14.00** Lunch
**14.00–15.00** **Addressing Medication Errors:** Common medication errors and ways to avoid these were addressed in a PowerPoint presentation. Time was allowed at the end of this session for students to ask questions	**14.00–15.15** **Student Presentation 2:** The final round of student presentations
**15.00–16.00** **Overview of Medication Contraindications:** Students were encouraged to view a pre‐recorded session with a pharmacist on: drug–drug interactions, drug–herbal interactions, and drug–disease interactions	**15.15–16.00** **Assessment:** The final assessment comprised 45 questions based on content covered over the two‐day workshop. Grades were automatically revealed to students through the virtual learning environment, Canvas (note: this assessment did not contribute to the students' final grade)
**Homework** Students were provided with two journal articles and an accompanying multiple‐choice quiz to complete before the second workshop	

### 2.3 Data collection

Following the workshop, an invitation to participate in the study was made on the students' virtual learning environment. All fourth‐year mental health nursing students who attended both days were invited to participate (*n* = 32, of which 27 were female and 5 were male). Demographics are presented in Table 2.

**TABLE 2:** Three focus groups by gender

Focus Group 1	3 females, 1 male
Focus Group 2	4 females
Focus Group 3	3 females, 1 male

Data were collected over the course of three focus groups, each comprising four participants. Focus groups were conducted by a member of the mental health nursing academic team who had not been involved in the workshop (two females; one male). A semi‐structured interview was used as a guide for the interviews conducted. Participants were asked questions about the relevance of the workshop to clinical practice, the timing of the workshop, aspects they most enjoyed, and suggestions for improvements. Interviews were conducted and recorded on MS Teams and lasted approximately 40‐min each.

### 2.4 Data analysis

Data were analysed using reflexive thematic analysis, a method of qualitative data analysis used to capture people's experiences of phenomena (Braun & Clarke, 2021). Immersion in the data took place by having two members of the team (JG; SW) listen to the recording numerous times. Data were coded by JG and reviewed by SW. Major themes were then generated from identified codes. These themes were discussed, refined, and reviewed before final write‐up.

Rigour was maintained by adhering to the principles outlined by Lincoln and Guba (1985). Credibility was enhanced by having experienced researchers lead focus groups. Furthermore, during data analysis, regular meetings between the research team were held, with disagreements resolved through consensus. An audit trail was maintained to track decisions. The steps involved in the research process are described in depth for transparency, thus enhancing dependability and confirmability. In order to enhance transferability, a purposive sample was used, with illustrative quotations from all focus groups provided (Lincoln and Guba 1985).

### 2.5 Ethics

Ethical approval was granted by the University's Social Research Ethics Committee. Students who had completed the workshop were invited to participate in a focus group and an information sheet on the study was provided. Consent forms were completed prior to participating in the focus groups. All data were stored according to General Data Protection Regulation guidelines. Data from the focus groups were transcribed by the research team. Once transcription was complete, recordings were destroyed. Anonymized data were stored on a password‐protected, encrypted laptop.

## 3 RESULTS

Three overall themes were identified: Developing Confidence around Medication Management, Reflections on Learning Gained from the Workshop, and Enhancing Awareness and Vigilance of Medication Errors.

### 3.1 Developing confidence around medication management

Participants spoke about their lack of confidence around medication. They felt that, over the years, they had developed confidence in their communication and interpersonal skills. However, they acknowledged knowledge deficits around medication.

I'm very confident in all the areas of nursing but when it comes to medication, I do have a bit of anxiety when I'm in front of the trolley, and confidence, not being familiar with the drugs (Focus Group 3, participant 1 [3.1])

It was suggested that learning about medication can be a daunting process. Several issues were highlighted, including trying to remember multiple trade and generic names, in addition to a lack of confidence giving or administering intramuscular injections (IMs). Owing to the volume of medication‐related information they were expected to retain, participants described feeling ‘overwhelmed’.

I find the, um, whole medication thing a bit overwhelming, um, I suppose, as well, there are so many different names for the same thing, which is, um, mind boggling (3.2)

I suppose students always have a bit of anxiety about giving IMs (1.2)

Some knowledge deficits only came to participants' attention during the workshop. Prior to the workshop, several participants felt that their knowledge around medication was sufficient; however, engaging in activities and having to answer questions revealed that further learning was warranted.

I really thought I knew my meds but then when he was asking me to identify them without knowing what they were, I struggled (1.3)

Following the workshop, participants observed an increase in confidence around medication management. Their knowledge, previously reported as limited, was improved, providing participants with an evidence‐base, perceived as fundamental to safe practice.

I found like it made me a lot more confident with what I was doing and it made me a lot more confident with doing IMs (2.1)

It's good to have the knowledge and the confidence behind it, yeah, to know what you are talking about and, um, and to be able to do it safely and effectively (1.2)

Consequently, communication with service users improved. Prior to the workshop, participants suggested that they were not comfortable speaking to service users about side effects and drug interactions. Following the workshop, owing to knowledge gained in these areas, participants felt more confident in addressing service users' concerns.

I'd know a bit more but, like, if the, um, service user asked me a question then I might be able to answer it more effectively […] with a service user even if you're asked a question about it you know if it's on the spot you can give them some answer and then, like, say, ‘I'll get you more information on it’, and you're able to give an answer to their question instead of, you know, like, ‘I don't have a clue’ (2.4)

### 3.2 Reflections on learning gained from the workshop

Participants commented positively on the interactive elements of the workshop. They appreciated that an active/participatory approach was used in favour of a passive/lecture‐type one. Through having students very much involved as active learners, concentration levels were sustained.

I enjoyed the interactive perspective where he put up the Kardexes [medication charts] and you spot the errors. I really found when you are more involved with the presentation that it was really good […]. I found that I learned more by engaging rather than by sitting back and listening (3.1)

The ‘medication image identification’ session was favourably evaluated, especially the practical elements. Participants were able to appreciate the importance of being able to identify medications, and they were able to directly relate this learning to their clinical placement experiences.

I found the pictures, d'ya know, the picture test that we got in the class. You'd be dispensing medication on placement, like, and you'd be able to remember, like, which one is which when you'd give ‘em, like, and it was good to teach you, like, um, what colour it is and when you're giving it, dispensing it, giving it out. It was really good to know (2.3)

Similarly, both ‘spot the error’ sessions were very easy to relate to clinical practice. Participants were easily able to identify how they could apply the learning gained from these sessions to everyday nursing scenarios.

I found the Kardexes [medication charts] really good and […] I was able to go in the next day and relate it so well to practice (1.3)

Participants highlighted both the practical and theoretical aspects of the intramuscular injection (IM) session. It was felt that using practical equipment – such as real needles – enhanced the authenticity of participants' learning experiences. However, participants also embraced the theoretical content, highlighting the importance of being aware of the theory that underpins decisions made in the clinical environment.

Then, like, demonstrating IMs and stuff – she had brought in the needles and stuff, and she was able to show us (2.1)

I thought it was very practical, because, like, I'm on the unit […] and we were doing IMs quite regularly, and so having that theory done, just, kind of in the thick of it was really, really handy (2.1)

It was noted that a great deal of knowledge was gained at the sessions on ‘addressing medication errors’ and ‘overview of medication contraindications’. Participants learned about the intricacies of certain medications. They also learned about the risks of some medications and the steps one needs to take in the event of making a medication error.

We had one lecture there and she went through lithium and Clozaril in depth (3.4)

It's something that could happen to anyone and, d'you know, like, the process I thought was interesting and she, um, spoke about […] what contributes to medication errors and, like, not reporting medication errors which I thought was really interesting (2.4)

It was reported that one of the highlights of the workshop was the student‐led presentations. As a result of having to present information to their peers, participants put extra effort into their individually assigned medications, learning about these in depth. Because participants were required to pay attention to one medication, important information relevant to these medications was retained.

We all had to do a presentation on medication. Everyone was given a medication and, like, I got Clopixol and so, like, d'you know, I have an in‐depth knowledge of Clopixol (1.1)

Every time I see that on a trolley, I don't have to think about it anymore, like. I dunno, like, it's in my head – I had to go away and learn about it and present it myself (1.2)

Aside from their own presentations, participants reported gaining invaluable insights into other medications from listening to their peers.

I might've only taking two or three things away from people's presentations, but you know, it's still just extra knowledge that's added on (1.3)

Participants also appreciated the facility to test their knowledge through the end‐of‐workshop examination. Crucially, because the examination was not formally assessed, participants did not feel under undue pressure. However, participants still felt the need to be challenged, and ultimately rewarded by receiving a high score.

It wasn't, like, part of the overall marks, and I think that's very important as well: to have quizzes that don't go into the overall marks, ‘cause it's like less pressure and it's more beneficial (3.2)

We had the questionnaire at the end, like, that did influence the learning, ‘cause we all knew we had an exam at the end of it so, like, I feel, like, you know, we probably were, like, even more, like, ‘oh I need to listen to all this ‘cause I want to do well in the exam’ (2.4)

It was noted that the workshop was well‐placed mid‐internship. Participants commented that they engaged in a similar learning experience in the second year. However, because medication management was not very relevant to their placement experiences at the time, they did not fully engage with this content, and were unable to recall what had been discussed.

They did something similar when we were in 2nd year. I couldn't really tell you anything from that presentation, I just wasn't able to relate it to practice at that time (1.3)

I think it was good to have it during the internship but before I was, um, like, ‘why are we having this now why didn't we have it before?’, but I think it makes sense: we're all in practice, we're all seeing the medications and doing everything, whereas if it was before we wouldn't have and we wouldn't have concentrated as much (2.2)

On the other hand, because they were due to qualify soon and would be directly involved in administering medication, participants were much more invested in a year four workshop. It was acknowledged that, in the absence of the practical ward and community‐based experience gained during internship, participants may not have engaged with the content of the workshop to the same level.

I thought fourth year was a great year to have it. […] Fourth year was the first year that, like, you're so hands‐on with it (1.4)

It would have been very overwhelming if we didn't know, like, a baseline amount of information, like, beforehand (2.4)

### 3.3 Enhancing awareness and vigilance of medication errors

A recurring concern expressed by participants was the unwelcome prospect of making medication errors. A self‐perceived lack of competence around medication coupled with uncertainty about the process of reporting an error resulted in stigma.

I feel like there is a stigma around it, that you would be afraid to, like, uh, say that you made one (1.4)

I think it's, like, a stigma that many nurses have, that if you unintentionally, like, make an error that you'll get in trouble (2.4)

Following the workshop, and having discussed the process of reporting medication errors, this stigma was reduced. Participants received clarity about the steps to follow in the event of making a medication error and developed their awareness about the importance of reporting even the most minor of errors.

I thought she was kind of emphasizing that if […] you made a medication error, it was really important, like, that you come forward because practice can't be improved. […] I thought it was interesting because, like, you know, definitely if something did happen, you know, you would report it like even if it's a minor matter or a near miss or whatever because these things matter (2.4)

This level of awareness was reported as apparent upon participants' return to the clinical environment. Post‐workshop, it was stated that changes to practice were made. Participants felt that the knowledge they gained allowed them to be more in tune with medication management, and that they could pay much more attention to developing their competence in this area.

Even now still when I'm giving out medication, I'm, um, kind of looking at it more intently that what I would've before the workshops. And I think that's something that I'm always going to do (1.3)

Due to the knowledge gained during the workshop, participants also reported being more vigilant in clinical practice. They were more aware of the side effects of certain medications and the reasons why medications are prescribed, which led to a cautious approach when administering medications. Furthermore, participants gained the confidence to question decisions made by others around prescriptions.

It made me realise […]with all the Kardex [medication chart] errors to just not to give out medications like candy, um, like, not even thinking what it's for, d'you know, why does the person need it and it could be the wrong dose or whatever and, like, sometimes doctors might prescribe double the dose or it might be two different medications doing the same thing and it might be, like, just to recognise that there can be like mistakes made, like, and that doctors aren't perfect (1.1)

Ultimately, it was felt that enhanced knowledge and awareness resulted in safer practice and better care for service users.

Even that is kind of promoting the safety of the service user that we are looking after, that kind of way, I think that's a good thing to see that direct line between, um, something that we have done in the classroom that has ended up in clinical practice, like, d'you know, so, like, anything we can learn or can add to our practice to keep our service users safe can, like, um, is, like, the ultimate goal really of learning (1.2)

Participants acknowledged that their learning would need to be developed further as they progressed with their careers. They viewed learning about medication as a lifelong process and indicated a determination to keep abreast of advancements and innovations in medication to maintain their competence.

It's just, like, constantly learning, like, even when we are five years qualified, like, we are constantly referring back to – making sure we know what all the medications are for, like, um, just not getting lackadaisical (1.1)

## 4 DISCUSSION

The aim of this study was to evaluate student nurse's experiences of an interactive medication workshop. Overall, participation in the workshop was identified as having a positive impact on the students' knowledge, competence, and confidence.

Student narratives in this study were consistent with the broader literature on medication management which identifies that nurses' levels of experience and knowledge around the administration of medicines are important determinants of clinical risk (Deheven et al., 2021). This, coupled with students' expectations about what is expected of them in practice, and the stigma attached to making a medication error, heightened student anxiety. The scaffolding of learning throughout undergraduate training and an understanding of a continuation of this process for qualified nurses did not appear to be widely acknowledged by students in this study (Benner et al., 2010; Morrissette, 2010; Muir‐Cochrane et al., 2018). Despite this, it was identified that the timing of the workshop, in the students' final undergraduate year, increased its salience, and, despite the unwelcome prospect of making a medication error, participation in the workshop helped to destigmatize the process of error reporting.

The design of the workshop, using a variety of skilled tutors and incorporating a multitude of interactive teaching strategies, such as simulation, provided a suitable pedagogical approach to address the theory–practice gap, something which presents an ongoing challenge for nurse education (Goodwin et al. 2019b). Here, the use of clinically based nurses using clinical equipment to deliver content on IM medications, an interactive/participatory approach, student presentations, and testing knowledge through a final informal exam were all considered by the students to be favourable approaches. This interactive approach to learning using a multitude of strategies is considered favourable to traditional ‘didactic’ instruction, can deliver more favourable educational outcomes, and increase satisfaction for students (Jones et al., 2018). Future strategies to enhance student nurses' knowledge around medication should consider the use of a variety of interactive teaching strategies.

Furthermore, the workshop was identified as a suitable means to address this widely recognized skills deficit at an important stage of students’ clinical training (Goodwin et al., 2019a). This increased the students' overall competence and confidence in terms of their perceptions of what it is to become a ‘safe’ practitioner. Vigilance was increased, not just through increased knowledge and bridging the theory–practice gap, but through providing students with the opportunity to reflect on current deficits in knowledge. In the context of students' desire to become ‘safe’, the creation of ‘cognitive dissonance’ during the workshop underpinned their motivation for further learning and also heightened their vigilance in practice (De Vries & Timmins, 2017). Students reported that this ultimately increased their confidence and competence. A phenomenon which was initially described as ‘daunting’ by students was now less so.

Communication between nurses and service users is also considered to be fundamental aspect of developing positive therapeutic relationships (Wright & McKeowan, 2018). The students in this study identified mutual benefits, in that the knowledge garnered in the workshop improved their interactions with service users. Whilst this workshop did not seek to address other organizational factors which may directly contribute to medication errors, such as short staffing and workloads (Härkänen et al., 2020; Kelly et al., 2017), students described experiences akin to increased ‘situational awareness’ of the clinical environment when engaged in processes relating to medication management (Walshe et al., 2019).

This study has limitations. Mental health student nurses from one university participated in this study; students from other universities may have had different opinions on the workshop. It should also be acknowledged that a small sample size was used in this study. Furthermore, although students reported improvements in their knowledge and confidence, the long‐term impacts of the workshop are not clear; future studies should consider adopting a longitudinal approach.

## 5 CONCLUSIONS

Findings from this study suggest that the right amount of information (content related to medication errors, intramuscular injection (IM) technique, medication chart interpretation, delivered over a 2‐day period) was delivered by the right people (academics and expert clinicians) at the right time (during internship placement), in the right way (through an experiential, practical, interactive workshop). At a time when mental health nurses are continuing to spend a large portion of their day administering medication coupled with the continued high rates of medication errors, evidence suggests that such workshops are warranted. Students reported that the workshop increased their confidence, competence, and vigilance in administering medication in clinical practice, and improved their ability to communicate to service users about medication.

### RELEVANCE FOR CLINICAL PRACTICE

The delivery of practical workshop‐based education can enhance mental health nursing students' confidence and competence around medication management. Such confidence and competence will foster safer practice, reducing the risks of medication errors. Additionally, given student nurses' enhanced knowledge from the workshop, there is the opportunity to enhance communication between service users and nurses about medication. This research has the potential to be used as a good practice model for the delivery of medication education to undergraduate mental health nursing students.

## FUNDING SOURCE

This research did not receive any specific grant from funding agencies in the public, commercial, or not‐for‐profit sectors.

## ETHICAL APPROVAL

Ethical approval was gained through the University Social Research Ethics Committee (Log 2021–086).

## Data Availability

The data that support the findings of this study are available on request from the corresponding author. The data are not publicly available due to privacy or ethical restrictions.
